# Induction of Cell Cycle and NK Cell Responses by Live-Attenuated Oral Vaccines against Typhoid Fever

**DOI:** 10.3389/fimmu.2017.01276

**Published:** 2017-10-12

**Authors:** Christoph J. Blohmke, Jennifer Hill, Thomas C. Darton, Matheus Carvalho-Burger, Andrew Eustace, Claire Jones, Fernanda Schreiber, Martin R. Goodier, Gordon Dougan, Helder I. Nakaya, Andrew J. Pollard

**Affiliations:** ^1^Oxford Vaccine Group, Department of Paediatrics, University of Oxford, NIHR Oxford Biomedical Research Centre, Oxford, United Kingdom; ^2^Department of Infection, Immunity and Cardiovascular Disease, University of Sheffield Medical School, Sheffield, United Kingdom; ^3^School of Pharmaceutical Sciences, University of São Paulo, São Paulo, Brazil; ^4^Microbial Pathogenesis Group, The Wellcome Trust Sanger Institute, Hinxton, United Kingdom; ^5^Faculty of Infectious and Tropical Diseases, Department of Immunology and Infection, London School of Hygiene & Tropical Medicine, London, United Kingdom

**Keywords:** typhoid, typhoid vaccines, functional genomics, vaccine immunity, Ty21a, NK cell, cell cycle regulation

## Abstract

The mechanisms by which oral, live-attenuated vaccines protect against typhoid fever are poorly understood. Here, we analyze transcriptional responses after vaccination with Ty21a or vaccine candidate, M01ZH09. Alterations in response profiles were related to vaccine-induced immune responses and subsequent outcome after wild-type *Salmonella* Typhi challenge. Despite broad genetic similarity, we detected differences in transcriptional responses to each vaccine. Seven days after M01ZH09 vaccination, marked cell cycle activation was identified and associated with humoral immunogenicity. By contrast, vaccination with Ty21a was associated with NK cell activity and validated in peripheral blood mononuclear cell stimulation assays confirming superior induction of an NK cell response. Moreover, transcriptional signatures of amino acid metabolism in Ty21a recipients were associated with protection against infection, including increased incubation time and decreased severity. Our data provide detailed insight into molecular immune responses to typhoid vaccines, which could aid the rational design of improved oral, live-attenuated vaccines against enteric pathogens.

## Introduction

*Salmonella enterica* serovar Typhi (*S*. Typhi) is the predominant cause of enteric fever, a non-specific febrile infection affecting between 9.1 and 17.8 million people globally each year (1–3). Transmission between humans occurs by ingestion of faecally contaminated food or water, after which *S*. Typhi invades the gut mucosa and may be taken up by phagocytic cells, before asymptomatic systemic dissemination to the reticuloendothelial system. Individuals presenting with typhoid fever develop non-specific symptoms including fever, abdominal pain and headache, and bacteremia ensues (4, 5). It remains unclear how innate immunity or the adaptive immune systems (following vaccination or prior infection) eradicates *S*. Typhi after infection. Detailed investigation of the molecular host responses to live-attenuated vaccines and interpretation in the context of responses seen after human exposure to virulent wild-type *S*. Typhi may provide useful insights informing future vaccine design.

Ty21a is an oral, live-attenuated typhoid vaccine that has been licensed in 1989 and administered to a large number of people (6), demonstrating a protective efficacy of 42–96% after three or four doses in clinical trials performed in typhoid-endemic regions (7–9). Ty21a has been attenuated by random mutagenesis, resulting in poor infectivity of and survival inside host cells such as macrophages (10–12). Other oral live-attenuated vaccine candidates in development include M01ZH09, which originates from the same Ty2 parent strain as Ty21a. By contrast to Ty21a, M01ZH09 has been attenuated by two specific deletions of the *ssaV* and *aroC* genes, designed to prevent systemic spread and rendering the strain replication deficient, while retaining macrophage infectivity (13). In clinical trials, this vaccine has been shown to be safe and immunogenic, leading to robust anti-LPS antibody responses (13–15), which previous studies suggested may be a correlate of protection following Ty21a vaccination (16). The precise mechanisms resulting in protective immunity against typhoid fever after oral vaccination are poorly understood, despite its administration to millions of people since licensure. While protection mediated by inactivated Vi-polysaccharide vaccines is likely to be anti-Vi antibody mediated, cell-mediated immunity (CMI) is induced by live oral vaccines may also play an important role (17, 18). In particular, *in vitro* stimulation of peripheral blood mononuclear cells (PBMCs) isolated from Ty21a recipients with *S*. Typhi antigens using autologous antigen-presenting cells indicated multiphasic CD8+ T cell responses over months following vaccination (19). These reports suggest that oral, live-attenuated vaccines act through a complex interaction including induction of protective T cell responses and humoral responses to a broader array of *S*. Typhi antigens.

While the aforementioned studies focused on CD8+ T cell responses, involvement of other cell types in responses to vaccination and infection is likely. Indeed, interrogation of responses following experimental challenge with *S*. Typhi Quailes strain has revealed activation of regulatory T (Treg) cells during acute disease and has highlighted a role for circulating monocytes and dendritic cells (DCs) in binding of *S*. Typhi within 24 h after challenge (20, 21). While B cells are also activated by Ty21a (22), the role of other cell types, including NK cells, in the development of protective responses following oral live-attenuated vaccination against typhoid remains unknown.

Using a controlled human infection model of typhoid fever, we recently evaluated the protective efficacy Ty21a and M01ZH09 (23, 24). In this study, we describe the transcriptional response and investigate how these may relate to the subsequent immune response detected in the volunteers. In this context, we explored the relationship of the transcriptome signature to the different humoral immune responses, post-challenge clinical findings, and downstream activation of cell-mediated immune processes, identifying profound differences in NK cell activation by the two vaccine strains tested.

## Results

### Differences Are Evident in the Transcriptional Patterns following Independent Immunization with Two Oral, Live-Attenuated Vaccines

**Figure 1:**
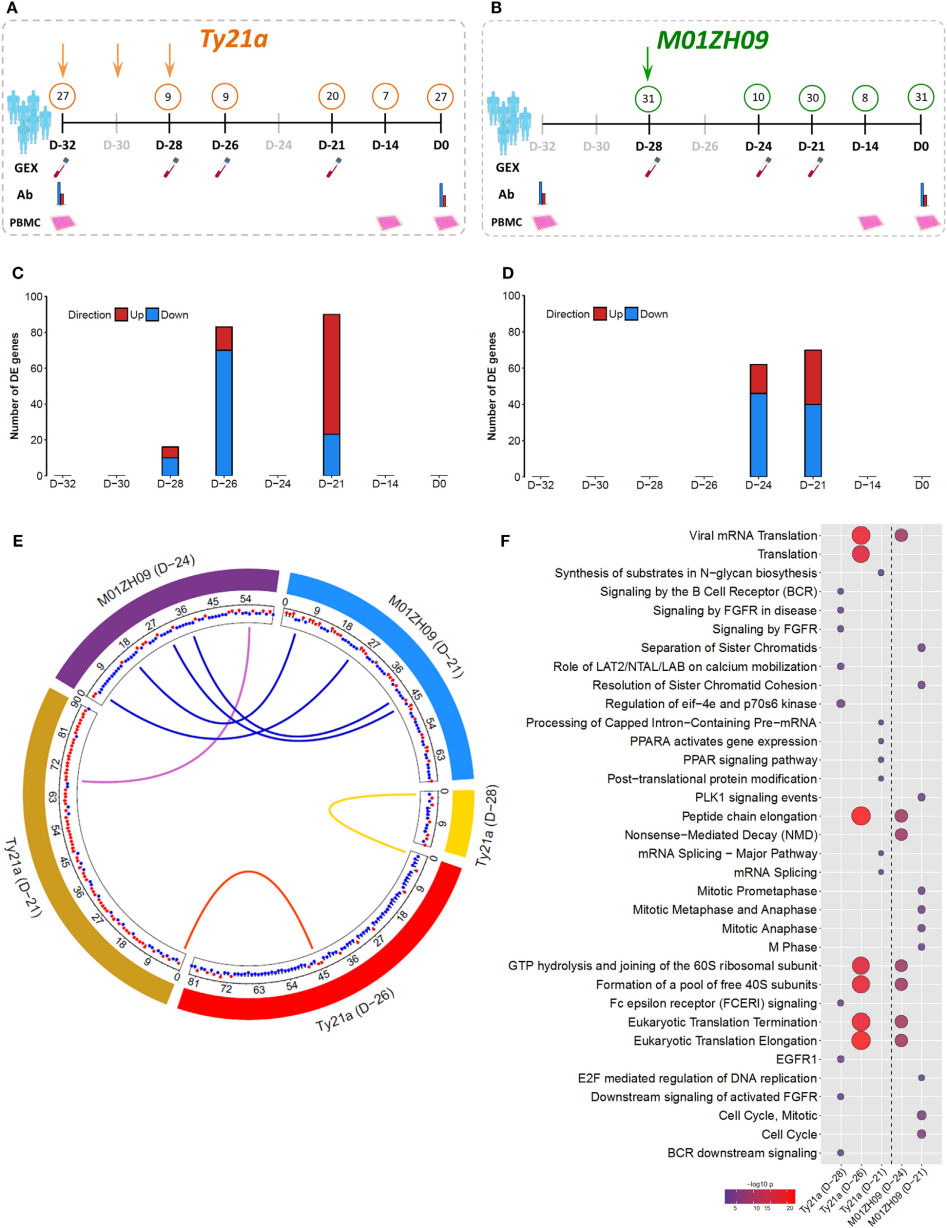
Gene expression (GEX) following oral, live-attenuated typhoid vaccination. **(A,B)** Study design and sample overview for Ty21a (orange; three doses administered at D-32, D-30, and D-28) and M01ZH09 (green; one dose administered at D-28). Numbers in circles represent *N* of which samples were taken at each time point/assay. Gray shaded time points indicated where no samples were taken. Samples were collected for GEX, antibody serology (Ab), and *in vitro* infection [peripheral blood mononuclear cell (PBMC)] analysis. **(C,D)** Number of differentially expressed (DE) genes over pre-vaccination baseline following Ty21a **(C)** and M01ZH09 **(D)** vaccination at time points after vaccination corresponding to panel **(A,B)**. **(E)** Circos plot indicating the overlap of DE genes between each vaccine arm and time point. Blue links: overlap between M01ZH09 time points. Purple links: overlap between M01ZH09 and Ty21a. Yellow and red links: overlap between Ty21a time points. Red and blue dots indicate up- or downregulation of each gene, respectively. **(F)** Significant pathways overrepresented by DE genes following Ty21a and M01ZH09 vaccination (increasing bubble size depicts increasing significance level).

To investigate host responses to live-attenuated oral typhoid vaccines, we vaccinated healthy adult volunteers with either three doses of the licensed vaccine Ty21a (*n* = 33), a single dose of the candidate vaccine M01ZH09 (*n* = 33), or placebo (*n* = 33), as part of a randomized, double-blinded, placebo-controlled trial (24). Gene expression (GEX) profiles were generated based on mRNA purified from whole blood before and at multiple time points following vaccination. The majority of measurements were using samples taken 7 days after completion of vaccines, i.e., at D-21, 7 days after the third dose of Ty21a and the first dose of M01ZH09 or placebo (Figures 1A,B).

At D-21, we observed moderate differential expression of the blood transcriptome in Ty21a vaccine recipients (90 genes) relative to baseline, and an even more modest differential perturbation in M01ZH09 vaccine recipients (70 genes, *p* < 0.001; Figures 1C,D). Due to the low level of perturbation in placebo recipients (29 genes; Figure S1A in Supplementary Material), subsequent analyses focused on Ty21a and M01ZH09 groups only. Transcriptional perturbation at early time points following vaccination (Ty21a: “D-28” and “D-26”; M01ZH09: “D-24”) was more pronounced in Ty21a recipients, potentially driven by the sequential doses of Ty21a in comparison with the single dose of M01ZH09. While we observed only one gene that overlapped both time points following Ty21a vaccination, more overlap was observed between D-24 and D-21 following M01ZH09 (Figure 1E). Interestingly, although Ty21a seemed to induce a similarly modest magnitude of transcriptional changes, it was significantly less immunogenic, as determined by anti-O9:LPS and anti-H responses 28 days following vaccination than was M01ZH09 (Figure S1B in Supplementary Material) (24).

To interrogate the signaling pathways stimulated by vaccination, we performed pathway overrepresentation analysis (ORA) of genes differentially expressed (DE) compared with baseline using the publically available database InnateDB (25). For Ty21a recipients, this analysis highlighted differential regulation of the pathways involved in the adaptive immune system and mRNA splicing. Furthermore, more general signatures of ribosomal subunit activity and translation elongation were highly overrepresented at the early D-26 time point and decreased at the D-21 time point. While ribosomal, and translation and elongation pathways were also DE soon after M01ZH09, we observed strong differential regulation of several pathways associated with cell cycle activity at 7 days following vaccination (D-21, Figure 1F). Although pathway analysis suggests differential activation of distinct molecular patterns compared between the vaccine arms, other similarities in expression patterns were observed, specifically at the earlier time points (D-28, D-26, and D-24) following vaccination.

### Inverse Activation of Cell Cycle and NK Cells following Oral Live-Attenuated Vaccination

To further interpret the overall molecular pattern underlying the GEX following live-attenuated oral vaccination against typhoid, we used Gene Set Enrichment Analysis (GSEA). GSEA is a complementary method to pathway ORA that instead of relying on threshold values to identify over- or underexpressed pathways or modules, employs a ranked list of genes to calculate enrichment scores for modules of interest (26). GSEA was performed using 14,302 genes ranked by log_2_ fold change(FC) against a conceptual framework of predefined blood transcriptional modules (BTMs) previously described by Li et al., with calculation of a normalized enrichment score (NES) and significance threshold for each module (27).

**Figure 2:**
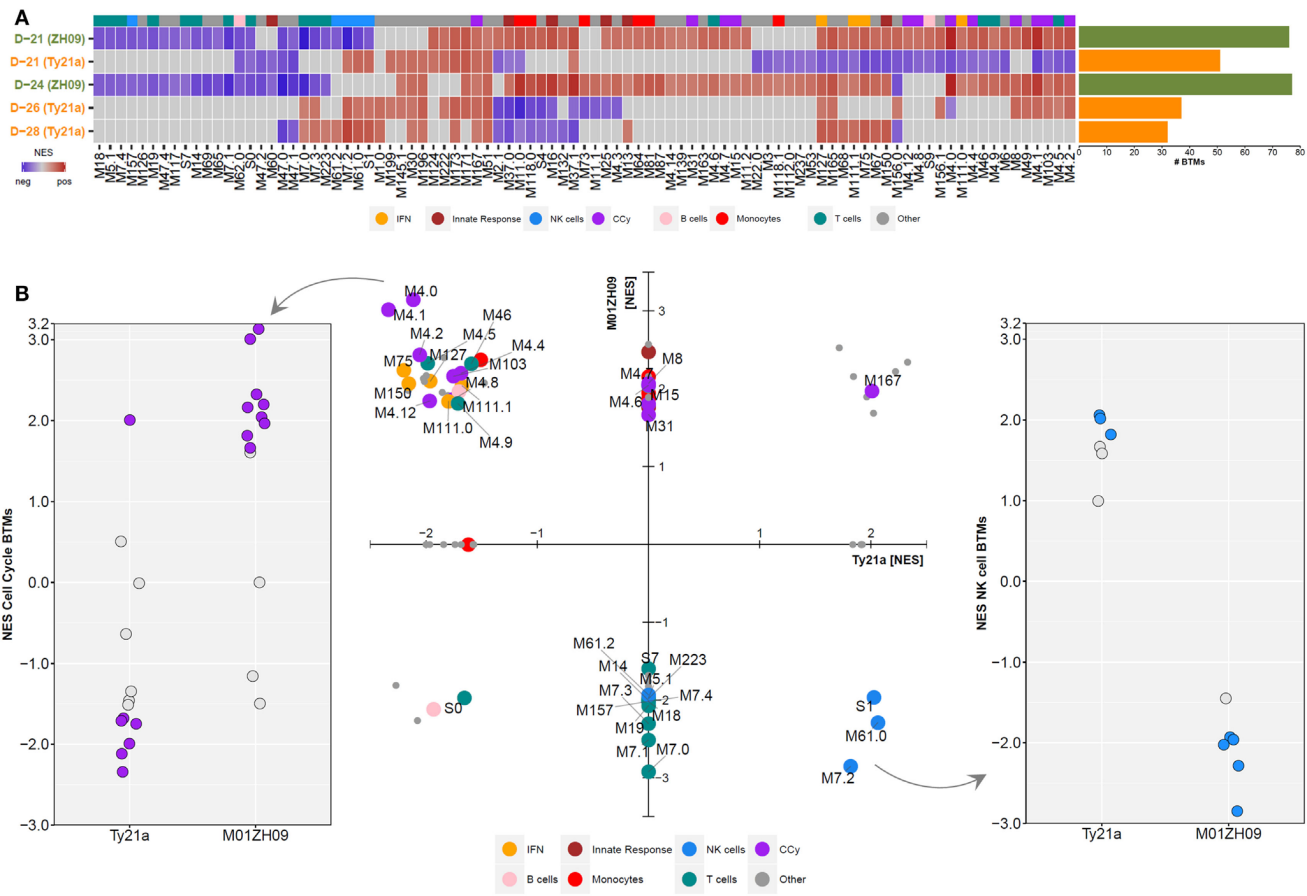
Gene Set Enrichment Analysis was performed at each time point following Ty21a and M01ZH09 vaccination. **(A)** Tile graph with significant blood transcriptional modules (BTMs) (adjusted *p* < 0.05) following vaccination (blue: negative enrichment; red: positive enrichment). Non-significant BTMs were set to normalized enrichment score (NES) = 0 (gray: not significant). Bar graph to the right indicates the number of significantly enriched BTMs at each time point/group. **(B)** NESs of BTMs at the D-21 time point following vaccination. Middle: scatter plot displaying NES of significantly enriched BTMs (adjusted *p* < 0.05) following either Ty21a (*x*-axis) or M01ZH09 (*y*-axis) vaccination. If a BTM was not significantly enriched in one of the two groups, the respective NES was set to 0. Colors represent different BTM categories. Dot plots depict enrichment scores of cell cycle (left) and NK cell (right) related modules. Solid dots: *p* < 0.05.

Shortly after vaccination with two (D-28) or three (D-26) doses of Ty21a, significant perturbation of several BTMs was observed (BH-adjusted *p* < 0.05) (Figure 2A). While some BTMs representing monocyte (M11.0 and M118.0) and innate response signatures (M.37.0, M16, and M25) were downregulated, several interferon (M127, M111.1, and M75), NK cell (S1, M61.0, M61.2, and M7.2), and T cell (M7.0, M7.3, and M223) modules were upregulated immediately following Ty21a ingestion (Figure 2A). Vaccination with M01ZH09 induced a higher number of BTMs at both time points measured (D-24 and D-21), compared with any time point after Ty21a vaccination. In addition, despite similarities in expression of some cell cycle and IFN modules, M01ZH09 elicited several responses at D-24 that contradicted those seen at the equivalent earliest time points after Ty21a receipt (D-26). These represented upregulation of monocyte (M11.0 and M118.0) and innate response (M37 and M16) modules and downregulation of T cell modules (M7.0, M7.3, and M223) in the M01ZH09 arm (Figure 2A).

Comparison of BTMs 7 days after completion of vaccination (D-21 in both groups) indicated further distinct differences between responses to the two oral vaccines. While positive enrichment of BTMs reflecting cell cycle control and monocytes was measured following M01ZH09, the opposite was observed (with negative or lack of enrichment) after Ty21a vaccination (BH-adjusted *p* < 0.05, Figure 2B—middle and left). By contrast, modules reflecting NK cell activity were upregulated after Ty21a and downregulated after M01ZH09 vaccination (BH-adjusted *p* < 0.05) (Figure 2B—middle and right). Of note, while most T cell modules were downregulated at D-21 following M01ZH09 receipt (and not significantly enriched following Ty21a), possible important differences were seen in three T cell-related modules. These modules [“mitotic cell cycle in stimulated CD4 T cells (M4.5),” “mitotic cell cycle in stimulated CD4 T cells (M4.9),” “cell division (stimulated CD4+ T cells) (M46)”] were upregulated in the M01ZH09 group but downregulated in the Ty21a group and represent cell division in CD4^+^ T cells indicating possible activation of specific T helper responses specifically following M01ZH09 vaccination. Overall, this group level GSEA has demonstrated interesting similarities and differences in the transcriptional response to two oral, live-attenuated typhoid vaccines. While transcriptional signatures of T cell responses appeared to be variable, a consistent difference in the expression of signatures representing NK cells was observed between the Ty21a and M01ZH09 arms.

**Figure 3:**
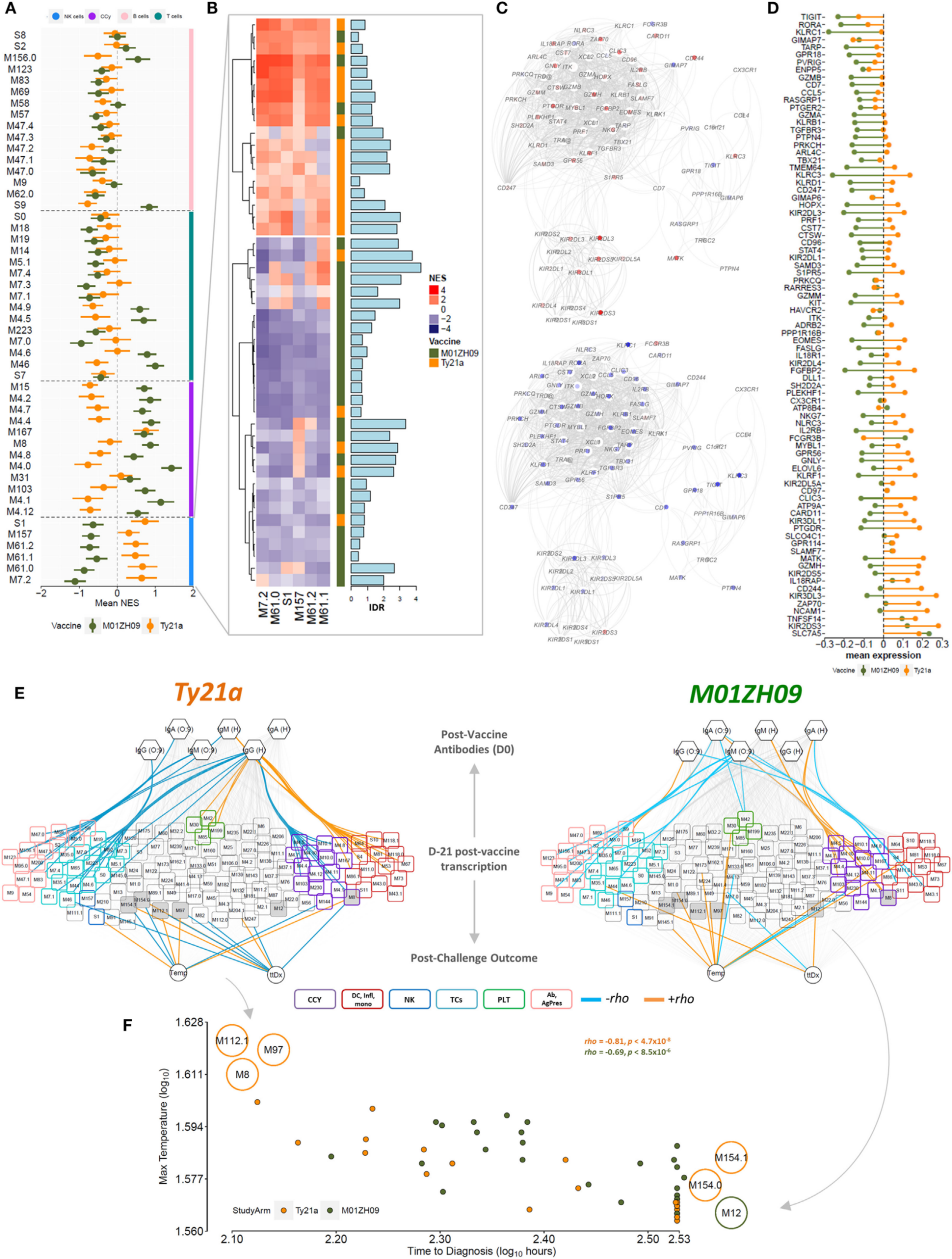
Single sample GSEA (ssGSEA) following vaccination at time point D-21. **(A)** Normalized enrichment score (NES) (mean + SEM) of blood transcriptional modules (BTMs) related to NK cells (blue bar), the cell cycle (purple), B cells (pink bar), and T cells (cyan bar) across all participants in the Ty21a (orange) and M01ZH09 (green) arm at time point D-21 after vaccination is plotted. **(B)** Tile graph representing the NES for all NK cell-related BTMs for each participant (red: positive NES; blue: negative NES). Color bar on the right represents the vaccine group membership. Bar graph along the tile graph signifies the heterogeneity within each participant (depicted by inter-decile range). **(C)** NK cell modules were combined into one network and superimposed with mean gene expression values at time point D-21 following Ty21a (top) and M01ZH09 (bottom). **(D)** Mean expression of genes within the NK cell-related BTMs in the Ty21a and M01ZH09 group at time point D-21 following vaccination. **(E)** Association networks. NESs derived from the ssGSEA at time point D-21 were correlated with antibody responses to vaccination and parameters reflecting outcome following challenge [temperature (Temp); time to diagnosis (ttDx)] in Ty21a (left) and M01ZH09 (right) recipients. BTM colors: purple: cell cycle. Blue: NK cells. Cyan: T cells. Red: inflammation, monocytes, and dendritic cells. Pink: antibodies, B cells, and antigen presentation. Green: platelet activation. Colors of edges represent significance level and Spearman’s rank correlation coefficients (orange: *p* < 0.05, rho > 0; blue: *p* < 0.05, rho < 0; gray: n.s.). Gray shaded nodes: BTMs associated with post-challenge outcomes. **(F)** Correlation plot of maximum Temp following challenge and ttDx. Modules indicated on the plot represent modules inversely associated with Temp and ttDx. Top left: positive and negative correlation with Temp and ttDx, respectively. Bottom right: positive and negative correlation with ttDx and Temp, respectively. Orange: Ty21a recipients; dark green: M01ZH09 recipients.

To further analyze the expression dynamics of modules among participants, we performed single sample GSEA (ssGSEA) at day 7 after vaccination (D-21). Plotting the mean expression (mean + SEM) of BTMs reflecting the cell cycle, NK cells, T cells and B cells highlights the differential expression pattern of cell cycle and NK cell modules between the vaccine groups (Figure 3A). Host immune responses to stimuli such as vaccines are known to vary between individuals as a result of both environmental and genetic factors (28, 29). To assess this variability, we plotted NK modules for each participant following vaccination and calculated the inter-decile range for each participant. This analysis highlighted the considerable variability in the response among the participants (Figure 3B). Overall, however, we observed consistent opposite expression of genes in the NK cell BTM including inhibitory and activating receptors including *KIR3DL3, KIR3DL1, KIR2DL3*, and *KIR2DL4* (Figures 3C,D).

### Cell Cycle Is Associated with the Magnitude of Humoral Immune Responses

Because ssGSEA provides enrichment scores for each BTM in each participant, these can be used to relate BTM expression to immunogenicity measurements (i.e., antibodies 4 weeks following vaccination) as well as parameters following challenge. Using Spearman’s rank correlation, we observed significant associations of cell cycle BTMs expressed at D-21 with antibody responses 28 days after vaccination, which were positive in M01ZH09 but negative in Ty21a recipients (Figure 3E). Moreover, we observed positive correlation of BTMs representing inflammation, monocytes and DCs and anti-H responses following Ty21a compared with M01ZH09 (Figure 3E). In addition, T cell-related modules were negatively associated with antibody responses following Ty21a vaccination. Of note, modules associated with B cell signaling and antigen presentation were negatively associated with serological responses to this vaccine. These data provide further insight into the modular response 7 days after vaccination, and the relationship to humoral responses suggesting that cell cycle BTMs may be predictive of humoral immunogenicity following oral live-attenuated vaccination.

### Modular Expression after Vaccination Is Associated with Delayed Onset of Disease following Challenge

Since this study was performed as part of a human challenge model, we selected two outcome measures following challenge to be correlated with enrichment scores at D-21 following vaccination. Time to diagnosis (ttDx) was delayed in participants receiving the M01ZH09 vaccine whereas there was no difference in maximum temperature (Temp) within 14 days following challenge in participants of both vaccine arms (24). This analysis showed that several modules were associated with post-challenge parameters, including “CD28 co-stimulation” (M12) and “enriched in cell cycle” (M167) following M01ZH09 vaccination. By contrast, following Ty21a vaccination, we observed BTMs that relate to transmembrane transport (M154.1), amino acid transport (M154.0), SMAD2/3 signaling (M97), E2F transcription factor (M8), and complement activation (M112.1) associated with post-challenge parameters. Associations with Temp and ttDx were inverse for several modules within respective vaccine arms (Figure 3E—gray nodes), showing a positive correlation with Temp but negative correlation with ttDx at the same time. Thus, M112.1, M97, and M8 expression following vaccination were positively associated with higher Temp, but shorter ttDx. By contrast, M12, M154.1, and M154.0 expression following vaccination was associated with lower Temp and longer corresponding ttDx (Figure 3F). These data underline the inverse relationship between disease severity markers (Temp) and ttDx of overt clinical disease in the challenge model and indicate possible protection indicated by module M154.0, M154.1, and M12 expression at time point D-21 following vaccination.

### Superior Capacity of Ty21a Compared with M01ZH09 to Activate NK Cells *In Vitro*

**Figure 4:**
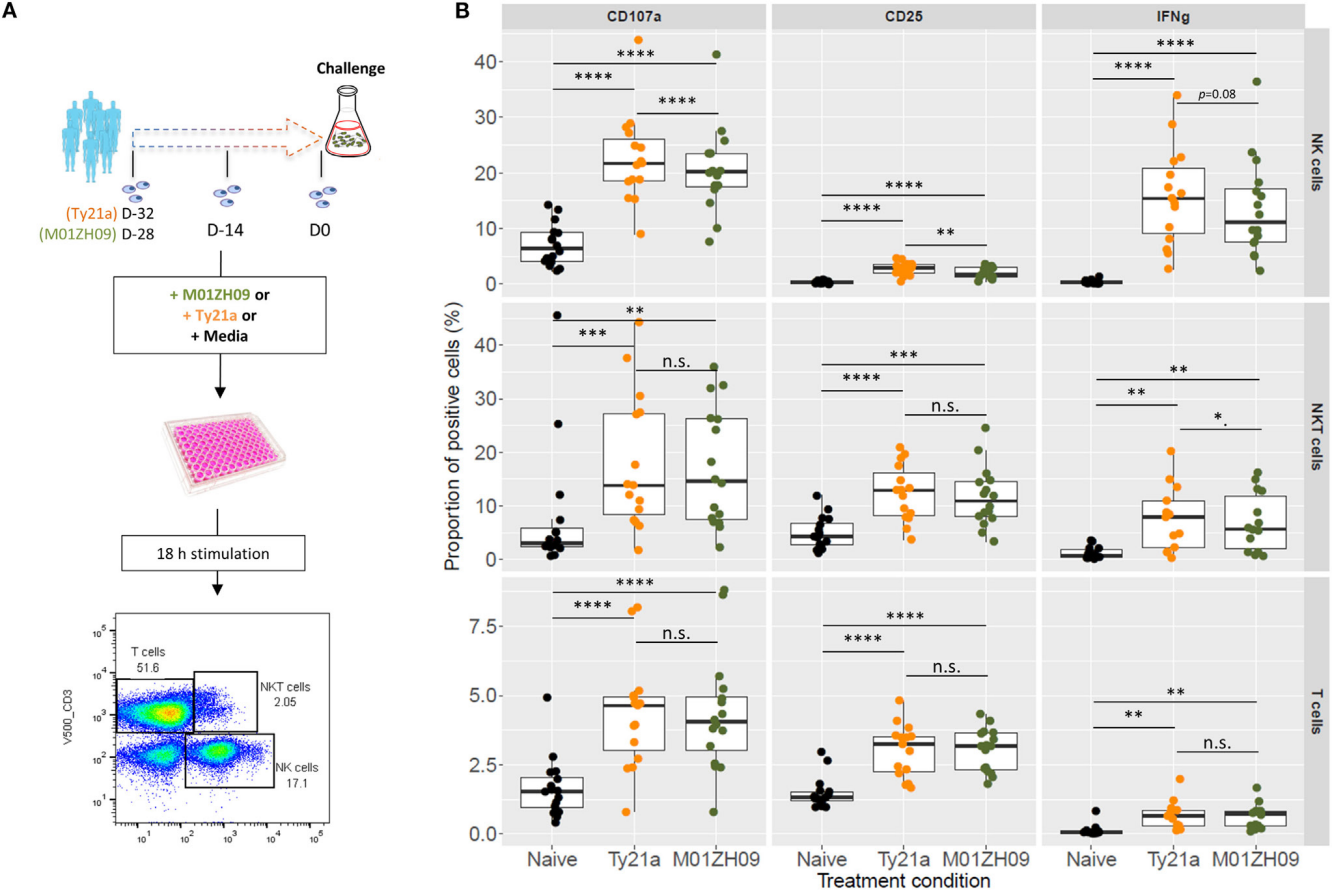
*In vitro* infection of peripheral blood mononuclear cells (PBMCs) from study participants. **(A)** Experimental design. PBMCs harvested from study participants at pre-vaccination (D-32: Ty21a; D-28: M01ZH09), 14 days (D-14), and 28 days after vaccination (D0, day of challenge) were infected *in vitro* with Ty21a (orange) or M01ZH09 (dark green). **(B)** Differential induction of activation markers on NK, T, and NKT cells following *in vitro* stimulation of PBMCs from vaccine-naïve volunteers with Ty21a (orange) and M01ZH09 (green). Multiplicity of infection = 0.1:1. Statistics: paired *t*-test: *p < 0.05; **p < 0.01; ***p < 0.001; ****p < 0.0001.

Given the major differential response in NK cell module activation between Ty21a and M01ZH09 vaccine recipients, we hypothesized that these vaccine strains might display differences in their capacity to activate NK cells *in vitro*. *In vitro* infection of PBMCs isolated from vaccine-naïve study volunteers (*n* = 16) before vaccination with either of the two vaccine strains (Figure 4A) indicated that Ty21a displayed greater capacity to induce surface expression of NK cell functional activation markers, including CD25 (*p* < 0.01) and CD107a (*p* < 0.0001), but not intracellular production of IFN-γ (*p* = 0.08), compared with M01ZH09. We also observed activation of these markers by Ty21a in CD3^+^ T and CD3^+^CD56^+^ (NKT and γδT) cell populations; however, no differences were observed between the two vaccine strains for CD107a and CD25 surface expression (Figure 4B).

**Figure 5:**
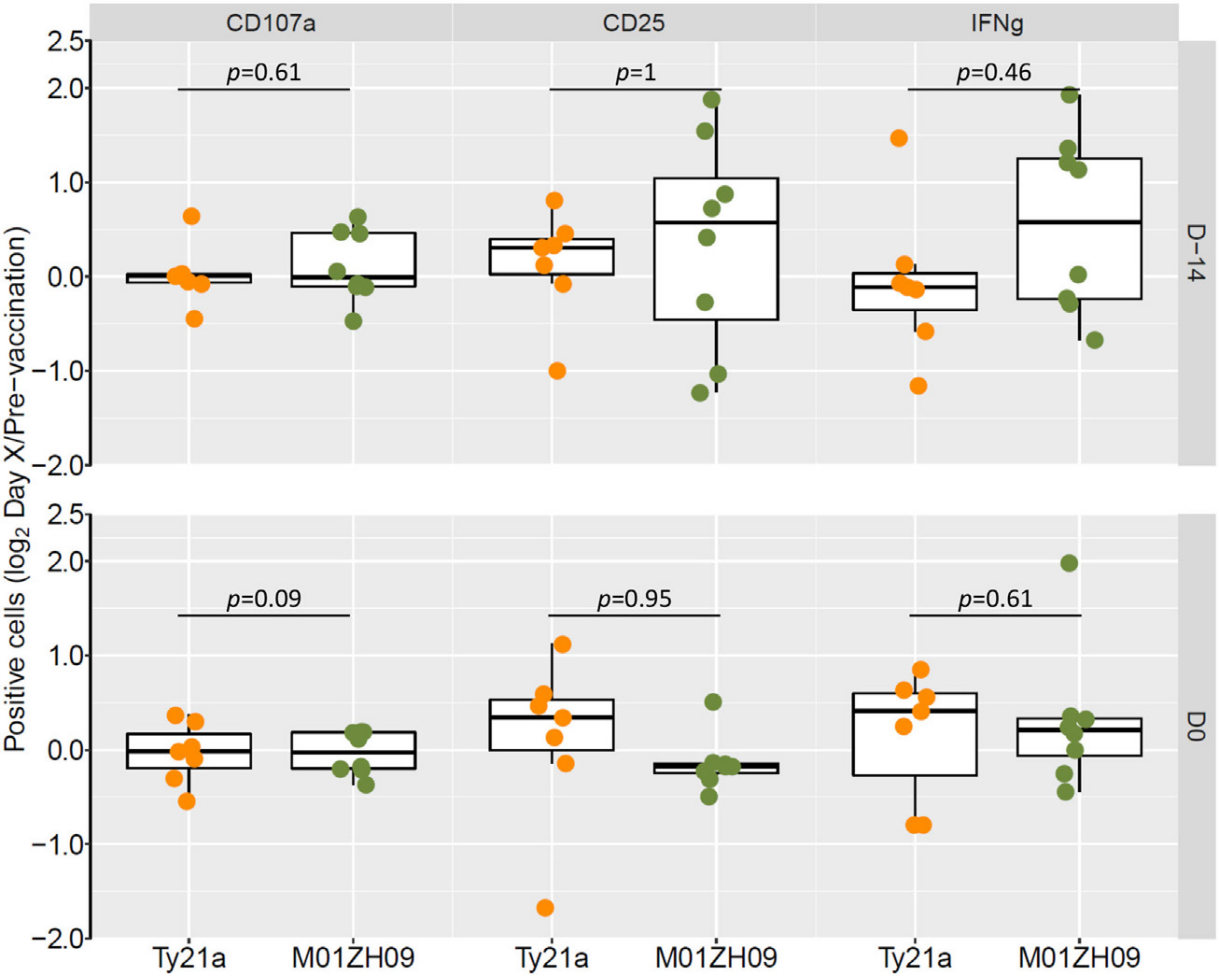
Autologous recall responses in peripheral blood mononuclear cells (PBMCs) from individuals post-vaccination. PBMCs harvested from study participants at 14 days (D-14) and 28 days after vaccination (D0, day of challenge) were infected *in vitro* with the autologous vaccine strain and NK cell responses measured. Fold-change increases of baseline responses are presented. Statistics: two-sided *t*-test.

Following vaccination with specific antigens, NK cells have been shown to mount a rapid effector recall response when re-exposed to the same antigen (30). To evaluate this phenomenon in the context of live-attenuated oral vaccines against typhoid, we reexposed PBMCs collected following vaccination (D-14 and D0) to the autologous vaccine strain in study participants. Overall, recall responses on exposure to the autologous vaccine strain did not differ at 14 days (D-14) or 28 days (D0) after vaccination with Ty21a or M01ZH09 (Figure 5). While some participants in the M01ZH09 arm appeared to display an enhanced response to the *in vitro* stimulation of post-vaccine PBMCs, the recall responses were not significantly increased compared with stimulated PBMCs collected from Ty2a vaccine recipients. Similar observations were made when PBMCs from participants were reexposed to the heterologous vaccine strain: no significantly increased recall responses were observed when PBMCs isolated from M01ZH09 and Ty21a recipients to Ty21a or M01ZH09, respectively (Figure S2A in Supplementary Material). Finally, we exposed the PBMCs to *S*. Typhi Quailes strain, the strain used in the subsequent challenge (24). Similar to the previous re-exposure experiments, a recall response to this strain was not observed after vaccination (Figure S2B in Supplementary Material).

## Discussion

Despite the administration of Ty21a to millions of humans, the immune responses induced following immunization with oral, live-attenuated vaccines against typhoid fever are still poorly understood. Here, we have generated detailed immune response profiles following two oral live-attenuated vaccines against *S*. Typhi, the three-dose Ty21a and the single-dose M01ZH09 vaccine. In addition to significant differences in humoral immunogenicity 28 days after vaccination, we observed differences in the incubation period and clinical severity following subsequent infection after experimental oral challenge (24). Our analyses uncovered differential transcriptional responses to these broadly similar oral vaccines, indicating a predictive relationship between transcriptional cell cycle signatures shortly after vaccination and humoral immune responses. Moreover, genes reflecting NK cell signatures were markedly DE underlining the differences in the immunological response induced by the vaccines and offering novel insight into possible mechanisms associated with protection.

Live-attenuated oral typhoid vaccines are thought to mimic much of the early infection process conferring protection by induction of the same pathways as natural infection but without systemic invasion and clinical symptoms. Despite originating from the same parent strain Ty2 (10, 13), Ty21a induced greater differential GEX than M01ZH09 representing distinct host responses with surprisingly little overlap in DE genes and transcriptional pathways between the vaccine strains. We observed differential regulation of the adaptive immune system (BCR signaling), RNA splicing, PPAR and EGFR1 signaling pathways after Ty21a vaccination. Alternative splicing patterns are part of transcriptional regulatory mechanisms involved in immune responses and in T cell activation (31, 32). This is further supported by the differential expression of PPAR signaling, which has been implicated in T cell differentiation (33) and linking the host’s lipid metabolism with differentiation of tissue-resident Treg cells (34). Notably, other patterns mostly representing ribosomal pathways were similarly expressed at the earlier time points in both Ty21a and M01ZH09 vaccine recipients; these were also the time points that were most comparable between the two arms when accounting for the differences in dosing schedule. To further interpret the GEX data, we used GSEA against predefined BTMs (27). In contrast to analysis at the gene level, M01ZH09 generated a greater modular response following vaccination. Because significant enrichment is based on a consistent positioning of module-associated genes at the top or bottom end of the ranked list, a higher number of significantly enriched BTMs suggest improved interpretability of the list and thus indicate a more coherent response. Importantly, the increased enrichment of the modular response following M01ZH09 is in agreement with the significantly stronger immunogenicity profile of M01ZH09 (24).

At early time points (D-28 and D-26) following vaccination with Ty21a, we observed upregulation of a small number of T cell-related BTMs, whereas these modules were downregulated or not significantly expressed early after M01ZH09 vaccination. By contrast, a different set of T cell-related modules were upregulated following M01ZH09 (D-24 and D-21) whereas downregulated or non-significantly expressed following Ty21a. These differences may reflect the differences in dosing regimen; however, the different type of these T cell modules may suggest that M01ZH09 induced signatures of proliferating T cells possibly aiding serological responses. Correlation analysis of BTM enrichment scores at D-21 with incubation period and disease severity following challenge highlighted protective associations (positive correlation with incubation period, negative correlation with Temp) of BTMs representing amino acid metabolism and transport (M154.0 and M154.1) after Ty21a vaccination. Alterations to the amino acid metabolism likely mirror the high metabolic demand during activation of adaptive immune responses, specifically T cells (35, 36). Indeed, *in vitro* studies have reported that in addition to antigenic T cell receptor stimulation, amino acids can provide the secondary activation signal for T cells through the metabolic regulator mTORC1 (37) and tempering this pathway can skew responses toward T cell memory (38). Intriguingly, modules correlating with incubation period and Temp were inversely related with both post-challenge parameters. This reflects the inverse relationship between ttDx and Temp, which is in keeping with previous findings of a similar relationship between incubation period and inflammatory signatures (39). In agreement with our data, Ty21a is known to induce multifunctional IFN-γ and TNF-α producing CD8^+^ T cells able to kill *S*. Typhi infected target cells within 2–8 days after vaccination (17, 19). These time points are comparable with our study with D-26 and D-21 equating to 2 and 7 days following the three-dose Ty21a regime, suggesting that the transcriptional signals measurable following Ty21a including enrichment of some T cell modules as well as amino acid metabolism are indicative of such T cell responses induced by this vaccine. Whether some of the signatures seen in response to three doses of Ty21a could be induced by similar, multiple dosing of M01ZH09 remains unknown. Finally, because Ty21a was more protective than M01ZH09 but induced weaker antibody responses, our results emphasize that oral live-attenuated vaccines are likely to confer protection predominantly through induction of CMI rather than antibody responses.

By contrast, transcriptional signatures of cell cycle activation were consistently positively enriched at D-24 (day 4) and D-21 (day 7) following M01ZH09 (including M4.0, M4.1, M4.2, M4.5, M4.7, and M103). Intriguingly, while we observed robust correlations of cell cycle modules with anti-O9:LPS and anti-H antibody responses 28 days after vaccination, B cell and T cell modules were negatively associated with serological responses. Since in whole-blood transcriptomics change in GEX can be related to changes in cellular composition of the blood, this observation possibly reflects the migration of cells to the lymphoid and/or gut mucosal tissues (40, 41) and thus a net downregulation of such modules. In turn, positive correlations between cell cycle modules and antibody responses 28 days after vaccination suggest proliferation of plasma cells and therefore predict serological immune responses, similarly seen with responses following acute typhoid fever (39). Moreover, such GEX profiles have been reported to be enriched 1, 3, and 7 days following influenza vaccination (42) and associated with anti-circumsporozoite protein antibody induction following RTS,S vaccination (43). These data in conjunction with reports that plasma cell responses generally peak around day 7 after vaccination with conjugate vaccines (PCV7 and MenC) suggest that transcriptional cell cycle signatures may provide an early peripheral blood marker of B cell generation (39, 44, 45).

We observed a striking induction of modules reflecting NK cell-related signatures following Ty21a vaccination. NK cells are a key cell type required for the production of IFN-γ, which is critical for protection in murine models of *Salmonella* infection (46). While some differences in modular responses may be attributable to the differences in dose regimen between Ty21a and M01ZH09, the NK cell signatures were remarkably consistent in its positive enrichment in Ty21a vaccinees even within 2 days of two or three doses. NK cells have been implicated in responses to live and attenuated vaccines; however, little is known of their specific role following vaccination, nor is it known whether live-attenuated oral vaccines stimulate and activate NK cells (30, 47). Using an *in vitro* infection model of PBMCs from naïve study volunteers stimulated with the vaccine strains, we indeed demonstrated a superior capacity of Ty21a to induce NK cell activation markers. This is of interest as NK cells can mount rapid recall responses upon re-exposure to the same vaccine antigen thus giving grounds for a role in protection by vaccination at the mucosal level (30, 47, 48). In our study, however, neither autologous nor heterologous re-exposure of PBMCs isolated from vaccine recipients at 14 and 28 days after vaccination showed recall responses following *in vitro* stimulation.

While we identified post-vaccination signatures associated with delayed onset of typhoid fever and decreased disease severity, these data should be interpreted with caution. The identification of surrogates of protection is not trivial as the attack rate in the challenge model is approximately 65%, thus complicating the identification of individual participants protected by the vaccine and the 35% who remain well throughout the 14-day challenge period. Therefore, other measures such as incubation period or Temp maybe more suitably for correlation with vaccine responses. The disparate responses triggered by the two vaccines may be based on one or more of several fundamental differences between the strains. First, the different attenuation methods resulting in altering capability to invade and survive inside host cells may be the reason for the differences in host responses observed. Second, an important difference is the dosing regimen as well as vaccine formulation (Ty21a enteric-coated capsule versus M01ZH09 oral suspension), which, although difficult to control in these *post hoc* experiments, may also result in divergent response profiles and overlapping kinetic of host cell stimulation. Our study design, however, prohibits the interrogation of whether M01ZH09 would increase activation of T cell-related pathways if administered as a similar, multiple three-dose regime. Three doses of Ty21a are likely to have marked effects on the mucosal environment and thus impact subsequent host responses. Moreover, non-vaccine-related aspects such as sampling time points following vaccination may confound the results observed (42, 43). Finally, unlike to Ty21a, M01ZH09 is (variably) able to express Vi due to the presence of the *viaB* operon, therefore possibly affecting host–pathogen interactions; however, Vi responses to the vaccine were low (24).

This study provides the first detailed account of the transcriptional host response following oral, live-attenuated vaccination against typhoid fever. Our results indicate important novel aspects of immune responses necessary for developing protection after oral vaccination against typhoid. Strategies to improve our understanding of these responses in better detail and their importance in combatting gut mucosal pathogens may provide important insights into the design of improved live-attenuated oral vaccines against enteric pathogens.

## Materials and Methods

### Study Design and Participants

A randomized, double-blind, placebo-controlled trial was performed at the Centre for Clinical Vaccinology and Tropical Medicine (Churchill Hospital, Oxford, UK) to assess the protective efficacy of a single dose of M01ZH09 (oral suspension and liquid formulation) compared with placebo against *S*. Typhi challenge 28 days after vaccination (NCT01405521), as previously described (24). Three doses of Ty21a (enteric-coated capsules) were given to a positive comparator arm. The trial was sponsored and monitored by the Oxford University Clinical Trials and Research Governance office, approved by NRES South Central—Oxford A (11/SC/0302) and conducted in accordance with the principles of the International Conference of Harmonization, Good Clinical Practice guidelines.

After study initiation (November 2011), an independent Data and Safety Monitoring Committee (DSMC) reviewed clinical and laboratory data relating to patient safety (months 1, 5, and 8) and interim unblinded analyses of vaccine efficacy (months 5 and 8). No changes to the study protocol or participant eligibility were recommended (24). Four weeks following vaccination, participants ingested 1.2 g/120 mL NaHCO_3_[aq] followed by 30 mL of 1–5 × 10^4^ CFU of *S*. Typhi Quailes strain using an ambulant study design (23). Following challenge participants were followed over 14 days, after which a 2-week course of antibiotics (ciprofloxacin, 500 mg twice daily, 14 days) commenced unless participants developed overt clinical disease, in which case antibiotic treatment commenced immediately. Criteria for typhoid diagnosis were either microbiological (≥1 positive blood culture collected after day 5) and/or clinical (fever ≥38°C sustained for ≥12 h) (23, 24).

### GEX Array Profiling

Peripheral venous blood (3 mL) was collected in Tempus blood RNA tubes (Applied Biosystems) at baseline (pre-vaccination controls) (*n* = 66) for all participants. For individuals randomized to receive Ty21a (three-dose schedule) blood was collected at 4 (D-28; *n* = 9) and 6 days (D-26; *n* = 9) after the first dose of Ty21a, and 7 days following the final (third) dose of the Ty21a vaccine (D-21; *n* = 20). For individuals randomized to receive M01ZH09 (single dose), blood was collected 4 days (D-24; *n* = 10) and 7 days (D-21; *n* = 30) following M01ZH09 vaccination (Figure 1A). Samples from the placebo arm were also collected at baseline (D-28; *n* = 8), 4 days (D-24; *n* = 9), and 7 days (D-21; *n* = 8) thereafter. Total RNA was extracted from all samples using the Tempus™ Spin RNA Isolation kit (Life Technologies). 50 ng of RNA was used for hybridization into Illumina HT-12v4 bead-arrays (Illumina Inc.) at the Wellcome Trust Sanger Institute, UK, and fluorescent probe intensities captured with GenomeStudio software (Illumina Inc.).

### Data Processing and Identification of DE Genes

Differential gene transcription was assessed by analysis of changes in transcript abundance in whole-blood samples collected during the study. Raw data were quantile normalized, background subtracted and log_2_-transformed using the Bioconductor suite in R (Version 3.0.1) (49). Transcripts represented by more than one probe on the array were collapsed using the probe with the highest mean expression for each gene using the *collapseRows()* function of the WGCNA package in R (50). The collapsed dataset was then filtered to include only probes significantly detected (*p* < 0.05) in 60% of all samples, yielding 14,302 genes. This preprocessed dataset was used for all subsequent analyses. Quality control was performed using the *arrayQualityMetrics()* function in R (51) and led to the exclusion of 14 samples. DE genes were determined by fitting a linear regression model including post-vaccination time point and date on which the array was run as covariates, and using participant number as blocking variable to ensure pairwise analysis using the *limma*-package in R (52). Contrasts were generated between each time point and pre-vaccination baselines.

### Gene Set Enrichment Analysis

Expression patterns following vaccination were analysed using GSEA in R. Gene lists, consisting of 14,302 genes derived as described in the Section “Data Processing and Identification of Differentially Expressed Genes,” were ranked by average FC and applied to the GSEA algorithm (53). Briefly, NESs of BTMs previously described by Li et al. (27) were calculated by evaluating the overrepresentation of module genes at the extremes (i.e., top or bottom) of the ranked gene list and nominal *p*-values calculated using a permutation test procedure (26). As this method can be applied to any ranked gene list, we adapted the analysis to calculate enrichment scores on a sample-by-sample basis (ssGSEA). In this analysis, per participant FCs at D-21 were calculated using each participant’s respective pre-vaccination baseline sample. Resulting lists was then ranked by FC and applied to the GSEA algorithm. BTMs, which were significantly enriched (*p* < 0.01) in ≥40% of the samples and only annotated BTMs were included in subsequent analyses.

### Antibody Responses

Vaccine responses were determined by measuring anti-LPS (O9:LPS) and anti-flagellin (H-antigen) IgM, IgA, and IgG antibodies using ELISA before and 28 days after vaccination, as previously described (24).

### Blood Mononuclear Cell (PBMC) *In Vitro* Infection Model

Peripheral blood mononuclear cells were isolated from study participants pre-vaccination (D-32 Ty21a; D-28 M01ZH09), and at 14 (D-14) and 28 (D0) days after vaccination and stored in liquid nitrogen. For further analysis, samples were thawed, seeded at 2 × 10^5^ cells/well in 96-well tissue culture plates in RPMI + 10% fetal bovine serum (FBS) and rested for 2–3 h. Frozen sucrose stocks of Ty21a and M01ZH09 grown to log-phase in Luria-Bertani broth were thawed and bacteria washed twice in phosphate-buffered saline before resuspension in RPMI + 10% FBS. Bacterial suspensions were added to PBMC *in vitro* at a multiplicity of infection (MOI) of 0.1:1. A low MOI was chosen to avoid maximal responses in order to interrogate increased recall responses following stimulation of PBMCs from vaccinated individuals. Gentamicin was added at 200 µg/mL 1 h post-infection to kill extracellular bacteria. Recombinant human (rh) IL12 (5 ng/mL) and rhIL18 (50 ng/mL) were used as a positive control. Following addition of bacteria or cytokines, PBMCs were cultured at 37°C, 5% CO_2_ for 15 h before addition of Golgistop and Golgiplug according to the manufacturer’s directions, before further culturing for 3 h.

### Flow Cytometry

Peripheral blood mononuclear cells were washed and stained with antibodies against cell surface markers CD3 (BD Biosciences), CD56 (BioLegend), CD107a (BD Biosciences), and CD25 (eBioscience). Subsequently cells were fixed and permeabilized (BD Cytofix/Cytoperm) before intracellular staining of IFNγ (BioLegend). Flow cytometric analysis was performed on a BD FACSAria flow cytometer collecting an average of 60,354 events (minimum: 50,000). Gating of cell populations was performed as follows: single events → live cells → lymphocytes → T cells (CD3^+^CD56^−^), NKT cells (CD3^+^CD56^+^), and NK cells (CD3^−^CD56^+^). Expression of surface markers CD107a and CD25 was determined for each cell population through comparison with FMOs run in each experiment. Expression of the intracellular cytokine IFNγ was determined for each cell population through comparison with a sample stained with an isotype control antibody run in each experiment (Figure S3A in Supplementary Material). For each cell subpopulation, at least 102 events were collected (Figure S3B in Supplementary Material). All data analysis was performed using FlowJo v10 (FlowJo, LLC).

### Statistical Analysis

To identify modules associated with immunogenicity and parameters following challenge, we performed Spearman’s rank correlation relating NESs for each participant and module to antibody responses 4 weeks following vaccination and maximum Temp within 14 days after challenge. For ttDx was defined as hours from challenge until antibiotic treatment commenced and participants not reaching the diagnostic criteria following challenge were censored at 337.2 h (14.05 days) after challenged. All statistical analysis was performed in R (version 3.2.4) (54) and association networks visualized in Cytoscape v3.2.5 (55).

## Data Deposition

Gene Expression Omnibus GSE100665.

## Ethics Statement

This study was carried out in accordance with the recommendations of the International Conference of Harmonization, Good Clinical Practice Guidelines, with written informed consent from all subjects obtained in accordance with the Declaration of Helsinki. The protocol was approved by the National Research Ethics Service South Central—Oxford A ethics committee.

## Author Contributions

The clinical trial and sample collection were performed by CB, TD, AE, CJ, GD, and AP. Experiments were conceived and performed by CB, JH, TD, MC-B, AE, CJ, FS, MG, GD, HN, and AP. Data analysis was performed by CB, JH, MC-B, FS, MG, GD, HN, and AP. The manuscript was prepared by CB and JH, and all the authors contributed to the final version.

## Conflict of Interest Statement

The authors declare that the research was conducted in the absence of any commercial or financial relationships that could be construed as a potential conflict of interest.
