# Supplementary figures and images for: Induction of Cell Cycle and NK Cell Responses by Live-Attenuated Oral Vaccines against Typhoid Fever
Source: Front Immunol. 2017 Oct 12;8:1276. doi: 10.3389/fimmu.2017.01276 (PMC5643418; doi:10.3389/fimmu.2017.01276)

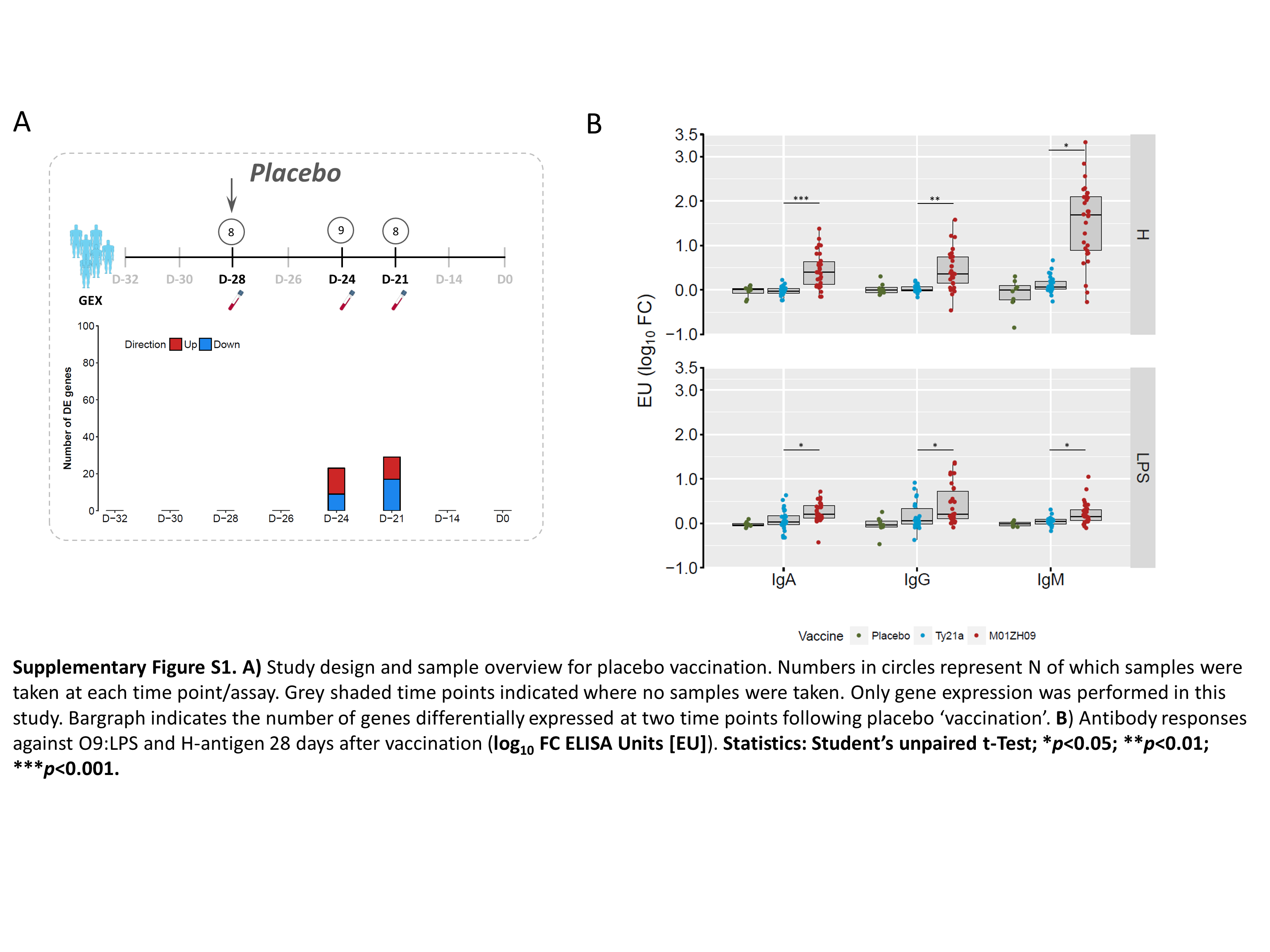

Supplement: Supplementary file 1 [file Image_1.TIF]

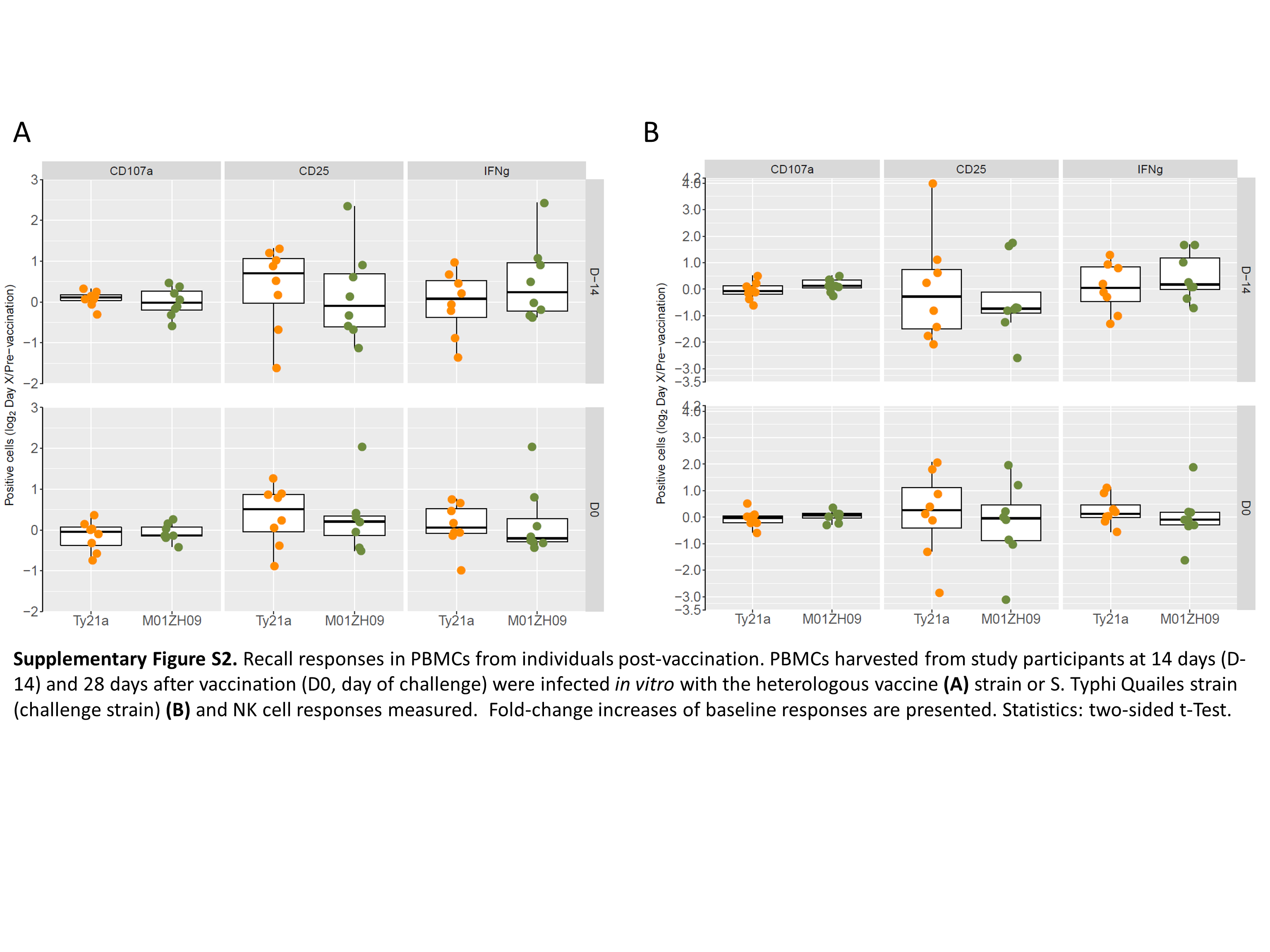

Supplement: Supplementary file 2 [file Image_2.TIF]

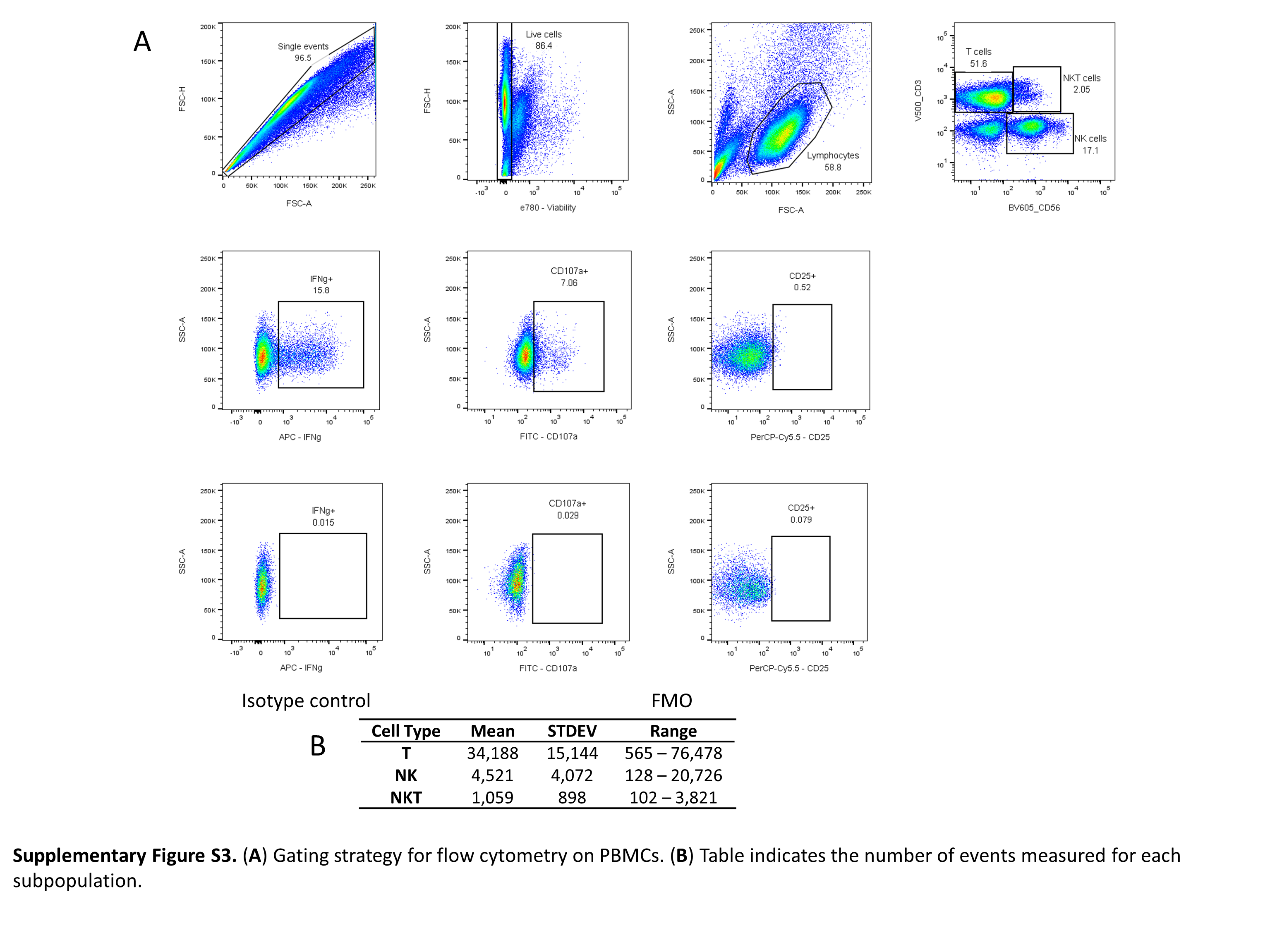

Supplement: Supplementary file 3 [file Image_3.TIF]
